# Sealing materials for post-extraction site: a systematic review and network meta-analysis

**DOI:** 10.1007/s00784-021-04262-3

**Published:** 2021-11-25

**Authors:** Massimo Del Fabbro, Grazia Tommasato, Paolo Pesce, Andrea Ravidà, Shahnawaz Khijmatgar, Anton Sculean, Matthew Galli, Donato Antonacci, Luigi Canullo

**Affiliations:** 1 Department of Biomedical, Surgical and Dental Sciences, Università degli Studi di Milano, Milan, Italy; 2 IRCCS Orthopedic Institute Galeazzi, Milan, Italy; 3 University of Genova, Genoa, Italy; 4 University of Michigan, Ann Arbor, USA; 5 Department of Periodontology, University of Bern, Bern, Switzerland; 6 Bari, Italy

**Keywords:** Alveolar ridge preservation, Coverage, Resorbable membrane, Non-resorbable membrane, Crosslinked membrane, Non-crosslinked membrane, Histomorphometric analysis, Systematic review, Network meta-analysis

## Abstract

**Abstract:**

**Aim:**

By means of a systematic review and network meta-analysis, this study aims to answer the following questions: (a) does the placement of a biomaterial over an extraction socket lead to better outcomes in terms of horizontal and vertical alveolar dimensional changes and percentage of new bone formation than healing without coverage? And (b) which biomaterial(s) provide(s) the better outcomes?

**Materials and methods:**

Parallel and split-mouth randomized controlled trials treating ≥ 10 patients were included in this analysis. Studies were identified with MEDLINE (PubMed), Embase, Cochrane Central Register of Controlled Trials, and Scopus. Primary outcomes were preservation of horizontal and vertical alveolar dimension and new bone formation inside the socket. Both pairwise and network meta-analysis (NMA) were undertaken to obtain estimates for primary outcomes. For NMA, prediction intervals were calculated to estimate clinical efficacy, and SUCRA was used to rank the materials based on their performance; multidimensional ranking was used to rank treatments based on dissimilarity. The manuscript represents the proceedings of a consensus conference of the Italian Society of Osseointegration (IAO).

**Results:**

Twelve trials were included in the qualitative and quantitative analysis: 312 sites were evaluated. Autologous soft tissue grafts were associated with better horizontal changes compared to resorbable membranes. A statistically significant difference in favor of resorbable membranes, when compared to no membrane, was found, with no statistically significant heterogeneity. For the comparison between crosslinked and non-crosslinked membranes, a statistically significant difference was found in favor of the latter and confirmed by histomorphometric NMA analysis. Given the relatively high heterogeneity detected in terms of treatment approaches, materials, and outcome assessment, the findings of the NMA must be interpreted cautiously.

**Conclusions:**

Coverage of the healing site is associated with superior results compared to no coverage, but no specific sealing technique and/or biomaterial provides better results than others. RCTs with larger sample sizes are needed to better elucidate the trends emerged from the present analysis.

**Clinical relevance:**

Autologous soft tissue grafts and membranes covering graft materials in post-extraction sites were proved to allow lower hard tissue shrinkage compared to the absence of coverage material with sealing effect. Histomorphometric analyses showed that non-crosslinked membranes provide improved hard tissue regeneration when compared to crosslinked ones.

**Supplementary Information:**

The online version contains supplementary material available at 10.1007/s00784-021-04262-3.

## Introduction

After dental extractions, a wound healing cascade occurs in fresh extraction sockets involving both hard and soft tissues. This sequence of biological events results in variable horizontal and vertical alveolar ridge resorption, both at the buccal and palatal/lingual aspects. This bone remodeling process may vary depending on individual local and systemic factors but especially affects the bucco-coronal horizontal thickness [1, 2] at anterior sites [3]. This phenomenon appears to be progressive and often results in esthetic and functional challenges during rehabilitation of partial or complete edentulism with dental implants [4].

To minimize the probability of needing bone augmentation at the time of implant placement, a wide variety of alveolar ridge preservation (ARP) materials have been described over the last decades. These include grafting with different biomaterials or biological agents with or without the placement of barriers to prevent ingrowth of soft tissues into the socket [5, 6, 7]. The biomaterial used to seal the socket should ideally inhibit epithelial and connective tissue ingrowth, stabilize the grafting material, and limit bacterial contamination [8]. In addition to resorbable and non-resorbable membranes, different biomaterials utilized for soft tissue augmentation such as autogenous free gingival grafts, dermal allografts, and collagen matrix xenografts have also been used to seal the socket [6, 9, 10].

Resorbable and non-resorbable membranes can be totally covered with a coronally advanced flap to achieve primary closure following the biologic principles of guided bone regeneration. However, this procedure inevitably leads to a change in the gingival architecture and position of the mucogingival junction. To avoid this, collagen membranes with different resorption periods may intentionally be left exposed to provide a transient barrier function [11]. The use of a dense polytetrafluoroethylene (d-PTFE) membrane with a low porosity also allows membrane exposure [12, 13]. Human placental allograft membranes [12], collagen sponges [8], and xenogenic non-crosslinked collagen matrices have also been utilized to seal sockets in this manner. Histologic studies of non-crosslinked collagen matrices in non-submerged or submerged environments revealed complete integration with mature mucosal and submucosal tissues and revascularization of the membrane after 3 months [14, 15]. Clinically, collagen matrices, when utilized as a barrier during ARP, are associated with a sufficient width of newly formed keratinized soft tissue [8, 16, 17]. Autogenous soft tissue grafts are another alternative but are associated with relatively high morbidity due to the fact they must be harvested from the palate.

However, at present, the influence of different biomaterials used to seal the socket on final ridge dimensions or histologic outcomes after ARP is not fully understood [11]. Therefore, the aim of the present systematic review and meta-analysis was to evaluate and compare the effect of different coverage materials (autologous palatal gingival grafts, resorbable membranes, and non-resorbable membranes) for ARP.

## Materials and methods

### Study registration

This review was conducted following the PRISMA guidelines (http://www.prisma-statement.org/). The review protocol was registered with PROSPERO (submission No. 196275).

The manuscript represents the proceedings of a consensus conference of the Italian Society of Osseointegration (IAO, https://www.iao-online.com).

### Patient, intervention, comparison, and outcome (PICO) question

The following focused questions were elaborated following the PICO format**:**

Population (P): patients receiving extraction and ARP

Intervention (I): ARP (using bone grafts or spontaneous healing) placing a biomaterial to seal the socket coronally (autogenous soft tissue grafts, allogeneic membranes (e.g., amnion-chorion membrane), resorbable collagen membranes (crosslinked [CM:Cross] or non-crosslinked [CM:NonCross], and non-resorbable membranes)

Comparison (C): ARP (using bone grafts or spontaneous healing) without placing a biomaterial to seal the socket (control, no sealing biomaterial)

Outcome (O): horizontal and vertical alveolar dimensional changes and percentage of new bone formation

### Focused questions

In patients undergoing tooth extraction and ARP:

1. Is the placement of a biomaterial over the extraction socket beneficial compared to healing without coverage in terms of horizontal and vertical alveolar dimensional changes and percentage of bone formation?
2. What is the relative efficacy of different available biomaterials for sealing sockets during ARP as compared to each other?

### Eligibility criteria

Randomized clinical trials with either parallel or split-mouth designs involving treatment of ≥ 10 patients (≥ 5 patients/group) that evaluated alveolar horizontal/vertical changes and/or newly formed vital bone after ARP with ≥ 2-month follow-up were eligible for inclusion. Included studies must have had the same bone grafting material or spontaneous healing used in both test and control groups, with the only difference being the type of biomaterial (membrane/autologous soft tissue grafting) used for coverage. Studies using the same coverage biomaterial in both test and control groups were excluded. Additionally, studies where both the graft and coverage materials differed between test and control groups were also excluded, due to the difficulty of statistically isolating the effects of these two variables. For studies with multiple treatments, only the data pertinent to the present review were considered. Lastly, studies evaluating soft tissue changes alone or volumetric changes were excluded. Studies including sites with complete socket wall destruction were excluded; studies including contentive defects (without one or maximum two resorbed/destroyed walls) were included.

### Search strategy

A literature search was carried out using electronic databases (MEDLINE (PubMed), EMBASE, Cochrane Central Register of Controlled Trials, Scopus), using an ad hoc search string that was adapted to each database: (((((((“tooth extraction”) OR “socket”) OR “alveolus”) OR “dental extraction”)) AND ((((((((((“bone grafts”) OR “biomaterials”) OR “autografts”) OR “collagen”) OR “cell therapy”) OR “platelet concentrates”) OR “alloplasts”) OR “allografts”) OR “xenograft”) OR “bioceramic scaffolds”))) AND (((((“alveolar ridge preservation”) OR “socket preservation”) OR “socket grafting”) OR “socket filling”) OR “ridge maintenance”). The electronic search was conducted up to 4th April, 2021. A hand search was also performed through pertinent dental journals. The reference lists of all identified RCTs and relevant systematic reviews were scanned for possible additional studies. Online registries were also checked (http://clinicaltrials.gov/;http://www.centerwatch.com/clinicaltrials/;http://www.clinicalconnection.com/). No language and date restrictions were made.

### Study selection

Two reviewers (LC, GT) independently screened the titles and abstracts of retrieved articles to identify eligible studies. The full text of all the eligible articles was obtained. Publications not meeting the selection criteria were excluded. Disagreements were resolved by discussion or by consulting a third reviewer (MDF). Two reviewers (GT, SK) assessed each study to confirm eligibility, and the reasons for exclusion were noted.

### Data collection

Relevant data to the study protocol (e.g., parallel or split-mouth design, flap or flapless technique, antibiotic prescription, presence or absence of the buccal wall, inclusion of smokers) were extracted from the included studies and reported in a predetermined datasheet.

### Primary outcome measures

1. Horizontal dimensional changes after ARP measured clinically or radiographically at the crestal level, at different vertical distances from the crest or from landmarks (adjacent teeth or implants).

2. Vertical dimensional changes after ARP measured clinically or radiographically at the crestal level or at the buccal and palatal/lingual aspects.

3. Percentage of newly formed bone evaluated through histomorphometric analysis of biopsies.

All of the above parameters were evaluated ≥ 2-month post-extraction.

### Risk of bias assessment

Two independent reviewers (LC, SK) evaluated the methodologic quality of included studies as part of the data extraction process. The risk of bias of included RCTs was assessed based on the following criteria adapted from the Cochrane Handbook for Systematic Reviews of Interventions: randomization method, concealed allocation of treatment, blinding of outcome assessors, completeness of outcome assessment reporting, and completeness of information on reasons for withdrawal by trial group. All criteria were scored as adequate/inadequate/unclear. The domain regarding blinding of participants and personnel (performance bias) was not considered because in ridge preservation neither the surgeon nor the patient can be efficiently masked to the bone graft material used, especially when the control treatment is self-healing. The reviewers contacted the authors of identified studies for clarification or to provide missing information as needed.

Studies were classified as low risk of bias (plausible bias unlikely to seriously alter the results) if all criteria were judged adequate; moderate risk of bias (plausible bias that raises some doubt about the results) if one or more criteria were considered unclear and none were considered inadequate; or high risk of bias (plausible bias that seriously weakens confidence in the results) if one or more criteria were judged inadequate. Disagreements between reviewers were resolved by discussion or by consulting with a third reviewer (MDF).

### Data analysis

Since the outcomes of ARP depend on many factors such as the graft material, surgical technique (e.g., flap/flapless, the use of a membrane), baseline condition of the socket (e.g., presence/absence of buccal or vestibular/lingual walls, wall thickness), post-operative management approach (e.g., antibiotic prophylaxis), and patient-specific response to treatment, a random-effects model according to DerSimonian and Laird was chosen to address variability. Both pairwise and network meta-analysis (NMA) were undertaken to obtain estimates for primary outcomes. The estimate of the effect of an intervention was expressed as mean differences (MDs) along with 95% confidence intervals (CIs). Heterogeneity among studies was assessed using Cochran’s test for heterogeneity and was considered significant when *p* < 0.1. The quantification of heterogeneity was estimated with *I*^2^ statistics. Substantial heterogeneity was considered when *I*^2^ > 50%. The software Review Manager (version 5.4, 2020; the Nordic Cochrane Center, the Cochrane Collaboration, Copenhagen, Denmark) was used for pairwise meta-analysis. Data from split-mouth and parallel group studies were combined using the generic inverse variance procedure. Meta-analysis was undertaken only when ≥ 2 studies with similar comparisons reporting the same outcome measures were found.

For NMA, the prediction intervals (PrIs) were calculated to predict future clinical effects by incorporating heterogeneity. Multidimensional ranking was also used to rank competing treatments. Inconsistency factor (IF) assess the presence of statistical inconsistency and netweight would assess, which comparison is affected more by high risk of bias studies. All analyses were done with Stata (version 16, StataCorp, College Station, TX) by one author (SK), with the commands xtgee, metan, mvmeta, network, and the routines from Chaimani et al. [18]. A two-tailed *p* value = 0.05 was considered statistically significant for hypothesis testing. Information regarding mean difference, SD, type of treatment, and number of subjects was collected from clinical studies. In situations where only one study was identified for a given comparison, the study was excluded because there would be network (geometry) disconnection, and no further analysis was possible. It was decided to divide data analysis according to the following categories, which are represented by the different sealing materials:

1. Control (no sealing)
2. Autogenous soft tissue grafts taken from the palate (CTG, connective tissue graft)
3. Allogeneic membranes (ACM, amnion-chorion membrane)
4. Resorbable collagen membranes (crosslinked or non-crosslinked)
5. Non-resorbable membranes

## Results

### Qualitative analysis of included studies

**Fig. 1:**
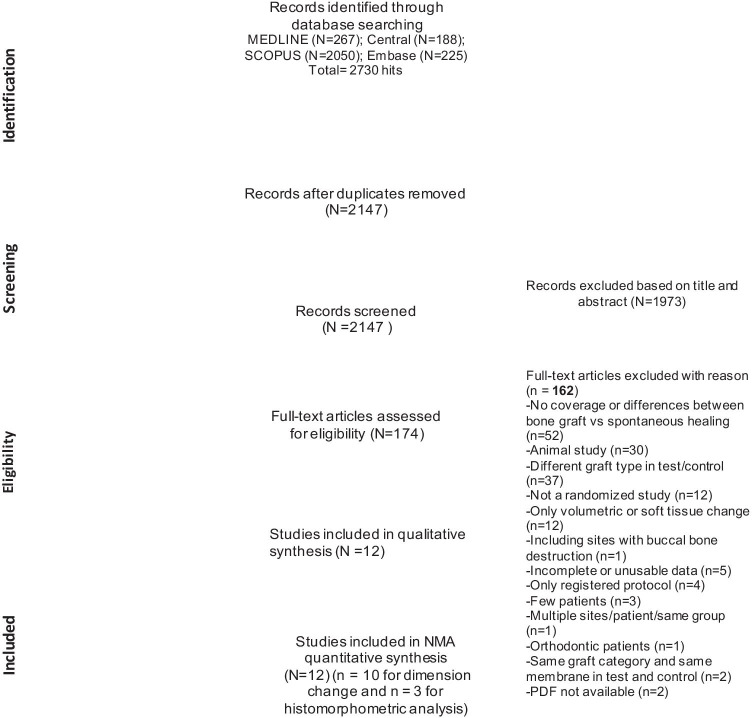
Flowchart of the study selection process

Fig. 1 provides a flowchart of the study selection process. The search strategy yielded a total of 2,147 items. After full-text evaluation of 174 eligible studies, 12 RCTs were included [8, 11–13, 16, 17, 19–24]. A total of 162 studies were excluded at this stage, and the reasons for exclusion were summarized in Fig. 1. Tables 1 and 2 describe the main features and outcomes of the included studies, respectively.

**Table 1:** Study included

Author	Study design	Country	Sponsor	Intention healing	Flap/flapless	Buccal wall	Antibiotics	Smokers incl. Y/N	n. tooth patients treated	Material control	ctr material type/name	Membrane Y/N	Coverage ctr	Material test	test material type/name	Membrane Y/N	Coverage test	Outcomes
Lim et al. [19]*	Parallel	South Korea	Y	Secondary	Flapless	Yes	Y	NR	33 (21 included in this review)*	SH	Blood clot	N	No membrane	XG	T1. DBBM-C + NBCM T2. DBBM-C	T1:Y T2:N	T1: NBCM➔ Resorbable collagen membrane (NCL)	Vertical/horizontal change/vital bone
Mandarino et al. [13]	Split-mouth	Brazil	N	First	Flap	Yes	Y	NR	20	SH	Blood clot	N	No membrane	SH	Blood clot	Y	Non-resorbable PTFE membrane	Soft tissue and hard tissue changes, vertical/horizontal change
Chang et al. [20]	Parallel	Korea	Y	Secondary	Flapless	Yes	Y	NR	42	XG	DBBM-C	Y	Resorbable collagen membrane (Bio-Gide) (CL)	XG	DBBM-C	Y	Resorbable Collagen membrane (Lyso-Gide) (NCL)	Vertical/horizontal change Cast analysis and CBCT
Hassan et al. [12]	Split-mouth	USA	Y	Secondary	Flap	Yes	NR	N	9	AG	FDBA + DFDBA	Y	Amnion-chorion membrane (ACM)	AG	FDBA + DFDBA	Y	Non-resorbable PTFE membrane	Vertical/horizontal change CBCT & histomorphometric analysis
Lim et al. [21]	Parallel	Korea	N	First	Flap	Yes	Y	Y <20/d	30	XG	DBBM-C	Y	Collagen membrane (NCL)	XG	DBBM-C	Y	Collagen membrane (CL)	Vertical/horizontal change
Natto et al. [8]	Parallel	Saudia	NR	Secondary	Flapless	Yes	NR	N	28	XG	Collagen sponge	Y	Resorbable collagen membrane	XG	Collagen matrix seal	Y	Resorbable collagen membrane (NCL)	Vertical/horizontal change
Arbab et al. [11]	Parallel	USA	NR	Secondary	Flap	Yes	Y	Y	24	AG+XG	Cancellous AG + buccal overlay XG	Y	Resorbable collagen membrane (NCL)	AG+XG	Cancellous AG + buccal overlay XG	Y	Non-resorbable PTFE membrane	Vertical/horizontal change
Parashis et al. [16]	Parallel	USA	NR	Secondary	Flap	Yes	Y	Y< 10/d	23	AG	FDBA	Y	Resorbable collagen membrane (NCL)	AG	FDBA	Y	extracellular resorbable collagen matrix membrane (NCL)	Vertical/horizontal change clinical & radiographic
Meloni et al. [17]	Parallel	Italy	N	Secondary	Flapless	Yes	Y	Y <10/d	30	XG	DBBM	Y	CTG	XG	DBBM	Y	Porcine collagen matrix (NCL)	Vertical/horizontal change CBCT
Perelman et al. [22]	Parallel	Israel	NR	First	Flap	Yes	N	N	23	XG	DBBM	N	No membrane	XG	DBBM	Y	Resorbable collagen membrane (CL)	Histomorphometric analysis
Engler-Hamm et al. [23]	Split-mouth	USA	Y	First Vs Secondary	Flap	Yes	Y	N	11	XG+AG	ABM/P-15+DFDBA + primary closure	Y	polyglycolic acid and trimethylene carbonate copolymer membrane	XG+AG	ABM/P-15+DFDBA + secondary closure	Y	Polyglycolic acid and trimethylene carbonate copolymer membrane	Clinical healing, histology
Lekovic et al. [24]	Split- mouth	Yugoslovia	NR	First	Flap	Yes	Y	NR	16	SH	Blood clot	Y	No membrane	SH	Blood clot	N	Resorbable membrane made of glycolide and lactide polymers	Clinical vertical/horizontal change (internal and external)

**Table 2:** Features of the study sample and outcomes for included studies

Authors	M/F	Age (mean, SD, range)	Tooth type/location (A/P)	Arch (max/mand)	Follow-up, months	n.teeth ctr treated	n.teeth ctr evaluated	n.teeth test treated	n.teeth test evaluated	Main conclusions	Complications
**Lim et al.** **[**19**]** *	16/5	54.36 +/− 9.91 (T1) 53.9 +/− 6.7 (T2)	Posterior (molar)	13 maxilla/8 mandible	4	T2: 10	T2: 10	T1: 11	T1: 10	Alveolar ridge preservation without primary flap closure in molar areas is effective in minimizing ridge resorption. There were no significant differences between test groups in clinical and histomorphometric measurements	0
**Mandarino et al.** **[**13**]**	10/10	46.2 +/− 15.6	Posterior	20 mandible	4	10	10	10	10	Ridge preservation using the d-PTFE membrane increased the formation of keratinized tissue. The membrane had no influence on the healing process	0
**Chang et al.** **[**20**]**	20/22	60.0 +/− 10.0 (T) 60.5 +/− 11.6 (C)	NR	21 maxilla/21 mandible	6	21	21	21	21	There were no significant differences between the ECM membranes in the changes in the dimension, width, and height of the extraction socket or the quantity of bone tissue	NR
**Hassan et al.** **[**12**]**	6/3	34–71 (54.88)	Anterior & premolars	14 maxilla/8 mandible	3	11	11	11	11	Intentionally exposed ACM is equally effective in ridge preservation compared to d-PTFE, however aid in reducing post-operative VAS scores and improved histomorphometric features	0
**Lim et al.** **[**21**]**	16/10	53.83 +/− 16.22 (T) 48.14 +/− 16.11 (C)	Anterior & premolars	Maxilla / mandible	4	15	14	15	12	The horizontal ridge alteration in ridge preservation did not differ significantly between using the non-cross linked and cross linked collagen membranes with collagenated bovine bone, but the vertical ridge alteration was more pronounced when using the cross linked membrane	Membrane exposure in the test group occurred in 47% and in the control group was 57% (8/14). Uneventful secondary healing was achieved in all cases
**Natto et al.** **[**8**]**	16/12	25 – 80 (55.4)	Single-rooted tooth (except lateral incisor)	23 Maxilla/5 mandible	4	14	14	14	14	Collagen matrix seal and CS, when combined with FDBA, significantly minimized ridge resorption in all dimensions and maintained buccal soft tissue thickness in sockets, however there was no significant difference between both groups	NR
**Arbab et al.** **[**11**]**	9/15	53 +/− 15 (CM) 52 +/− 15 (PTFE)	Anterior & premolars	22 maxilla/2 mandible	4	12	12	12	12	No significant difference (*p* > 0.05) was found in horizontal and vertical dimensional change between CM and d-PTFE group. Moreover, the percent vital bone was similar and not significantly different between both groups	0
**Parashis et al.** **[**16**]**	12/11	35–65 (54.2) (CM) 36–68 (54.8) (ECM)	Incisors & premolars	21 maxilla/2 mandible	4	11	11	12	12	No significant differences were observed between CM and ECM group in clinical and radiographical soft tissue and hard tissue changes. However, significant correlations for changes in gingival thickness (*p* = 0.001) and crestal bone width (*p* = 0.002) with pre-operative buccal plate thickness were observed	1 pt in the CM group presented post-surgical infection with exfoliation of the graft
**Meloni et al.** **[**17**]**	12/18	26–72 (48)	Anterior	Maxilla	12	15	15	15	15	Significant correlations for changes in gingival thickness (*p* = 0.001) and crestal bone width (*p* = 0.002) with pre-operative buccal plate thickness were observed	0
**Perelman et al.** **[**22**]**	7/16	26–68	Single-rooted tooth	18 Maxilla/5 mandible	9	11	11	13	12	Bone area fraction was found significantly higher in group augmented with DBBM and collagen membrane (*p* < 0.05)	0
**Engler-Hamm et al.** **[**23**]**	4/7	20–57 (41.09)	Posterior	Maxilla	6	11	11	11	11	Ridge preservation without flap advancement preserves more keratinized tissue and has less post-operative discomfort and swelling	NR
**Lekovic et al.** **[**24**]**	10/6	52.6 +/− 11.8	Anterior & premolars	Maxilla/mandible	6	16	16	16	16	Alveolar ridge preservation with bioabsorbable membrane made of glycolide and lactide polymers showed significantly less loss of alveolar bone height, more internal socket bone fill, and less horizontal resorption of the alveolar bone ridge , compared to sites without membrane	0

Overall, 321 sockets (159 in the control group and 162 in test groups) were treated, and 312 sites were evaluated (156 in the control group and 156 in test groups). In two studies, the control and test sockets were left to heal spontaneously [13, 24]. The follow-up duration ranged from 3 to 12 months (mean 5.5 months). One study included sockets in which the buccal wall was absent [22]. Antibiotics were prescribed in 10 studies.

The scenarios presented in the included studies were the following:

1. Same type of bone graft with and without a membrane (Lim 2019, Perelman)
  43 sites in 43 points
2. Self-healing (SH) with and without a membrane (Mandarino, Lekovic)
  52 sites in 36 points
3. Same type of bone graft with different membranes in group 1 and 2 (Chiang, Hassan; Lim 2017; Natto, Arbab, Parashis, Engler-Hamm)
  187 sites in 168 points
4. Same type of bone graft + autogenous soft tissue graft (CTG) vs resorbable non-crosslinked membrane (Meloni)
  30 sites in 30 points

There was a large degree of variability between studies in terms of (a) techniques and materials used; (b) outcomes investigated; (c) sites of tooth extraction; (d) timing of evaluation; (e) reason for extraction; (f) inclusion of compromised sockets; (g) use of antibiotics; and (e) flap vs. flapless extraction.

Additionally, some studies measured overall changes in the vertical or horizontal dimensions [19, 24], others distinguished between buccal and lingual/palatal measurements [12, 13], others estimated dimensional changes at the crestal level [11], and certain studies evaluated changes at different vertical levels apical to the crest [8, 11, 16, 17, 19–21, 23]. In the present review, the mean dimensional values were calculated and used to compare results of the 12 included articles.

The time interval between ARP and the post-surgical assessment varied between 3 and 12 months (see Table 2 for more details).

The techniques used to measure vertical and/or horizontal alveolar changes were different between studies, involving direct radiographs (either 2D or 3D) [8, 12, 16, 17, 19–21], digital scans of casts obtained by impressions [20], or clinical measurements using calipers [11, 13, 22]. Moreover, the sites of tooth extraction were variably distributed both in the mandible and in the maxilla and both in the anterior and posterior areas (see Table 2 for more details).

This systematic review and meta-analysis failed to draw strong conclusions mainly due to substantial heterogeneity among the 12 included studies, similar to results reported by a previous study on this topic [25].

### Risk of bias analysis

**Fig. 2:**
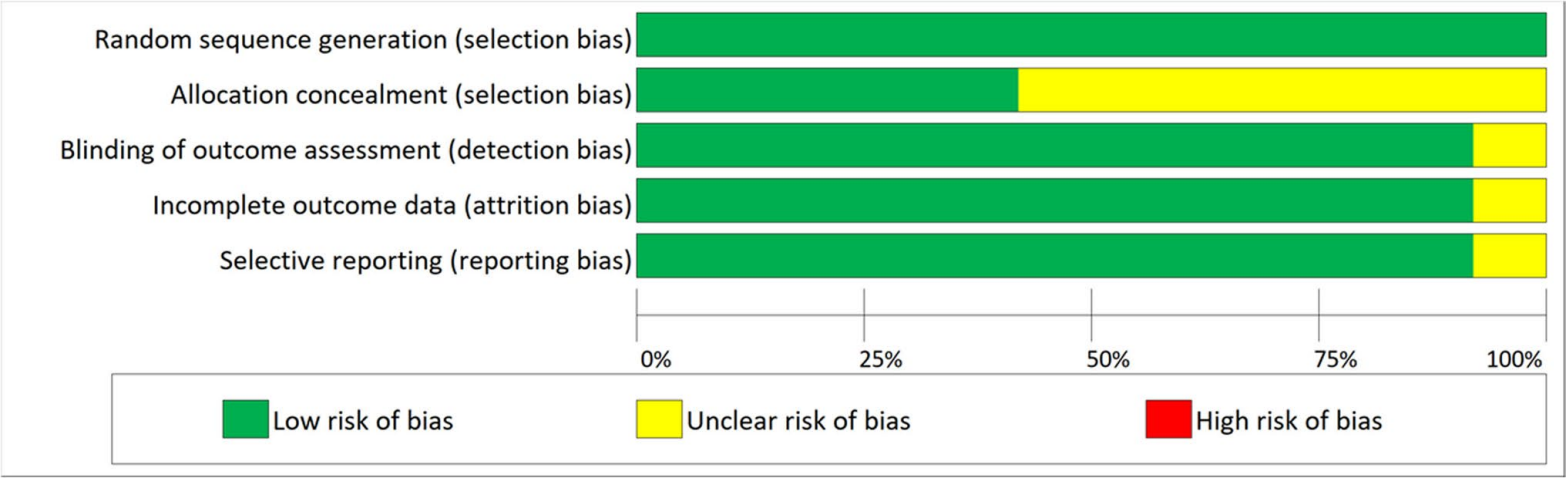
Risk of bias graph

Five studies were associated with a low risk of bias [8, 12, 16, 17, 21], and 7 were associated with a moderate risk [11, 13, 19, 20, 22–24] (Fig. 2).

### Pairwise meta-analysis

The comparison “resorbable membrane vs no membrane” included three studies [20, 22, 24]. Lim et al. (in groups 2 and 3 of their study) [19] and Perelman et al. [22] placed xenograft in the sockets, while Mandarino et al. [13] and Lekovic et al. [24] left the sockets unfilled. However, only Lim et al. [19] and Lekovic et al. [24] measured dimensional changes, while Perelman et al. [22] provided histomorphometric results. The use of a collagen membrane was associated with significant preservation of both horizontal (*p* < 0.00001) (Supplementary Fig. S1A) and vertical (*p* = 0.03) (Fig. S1B) dimensions. In both cases, heterogeneity was insignificant. Figure S2 shows pairwise meta-analysis for histomorphometric data. The use of collagen membranes resulted in significantly higher new bone formation (*p* = 0.003) compared to no membrane. No heterogeneity was detected.

Regarding other comparisons between sealing biomaterials, crosslinked vs. non-crosslinked collagen membranes were evaluated by two studies [20, 21]. Both studies used collagenated deproteinized bovine bone as the grafting material. The meta-analysis showed a significant advantage for non-crosslinked over crosslinked collagen membranes for both horizontal (*p* = 00005) (Fig. S3A) and vertical (*p* = 0.02) (Fig. S3B) dimensional changes. No heterogeneity was detected.

No other comparison was possible because there were not at least 2 studies which presented similar comparisons reporting the same outcome measures.

### Network meta-analysis

Allogeneic membranes were not considered in the NMA because only one included study used it [12]. Also, the effect of primary vs secondary healing could not be assessed, as it was heterogeneously reported, and comparisons were unfeasible (Table 3).

**Table 3:** Grading of network meta-analysis evidence

		Direct evidence		Indirect evidence		Network evidence	
		Odds ratio (95% CI)	Quality of evidence	Odds ratio (95% CI)	Quality of evidence	Odds ratio (95% CI)	Quality of evidence
Horizontal	CM V CM:Cross	0.42 (−2.65, 3.49)	Low	−1.09 (−4.79, 2.61)	Moderate	1.51 (−3.30, 6.33)	Moderate
	Con V Nonresorbable	−0.08 (−2.14, 1.97)	Low	1.42 (−2.92, 5.78)	Moderate	−1.51 (−6.33, 3.30)	Moderate
	CM:Noncross V Auto	0.04 (−0.93, 1.01)	Low	−3.56 (−4.66, −2.46)	High	3.60 (2.13, 5.07)	High
	CM:Cross V Auto	−3.25 (−4.19, −2.30)	High	0.35 (−0.77, 1.48)	Low	−3.60 (−5.07, −2.13)	High
	CM:Cross V CM:Noncross	0.30 (−0.90, 1.51)	Low	−1.65 (−4.07, 0.76)	Moderate	1.96 (−0.74, 4.66)	Moderate
	Nonresorbable V CM:Noncross	0.79 (−1.96, 3.56)	Low	−0.71 (−4.66, 3.24)	Low	1.51 (−3.30, 6.33)	Moderate
Vertical	Con V CM:Cross	0.36 (−2.30, 3.04)	Low	0.46 (−2.26, 3.19)	Low	−0.09 (−3.19, 3.72)	Low
	Con V Nonresorbable	−0.70 (−1.93, 0.53)	Low	−0.79 (−4.40, 2.81)	Low	0.09 (−3.72, 3.91)	Low
	CM:Cross V CM:Noncross	0.46 (−0.75, 1.68)	Low	0.37 (−3.24, 3.99)	Low	0.09 (−3.72, 3.91)	Low
	Nonresorbable V CM:Noncross	−0.70 (−2.80, 1.40)	Low	−0.60 (−3.79, 2.57)	Low	−0.09 (−3.91, 3.72)	Low

#### Vertical outcomes

**Fig. 3:**
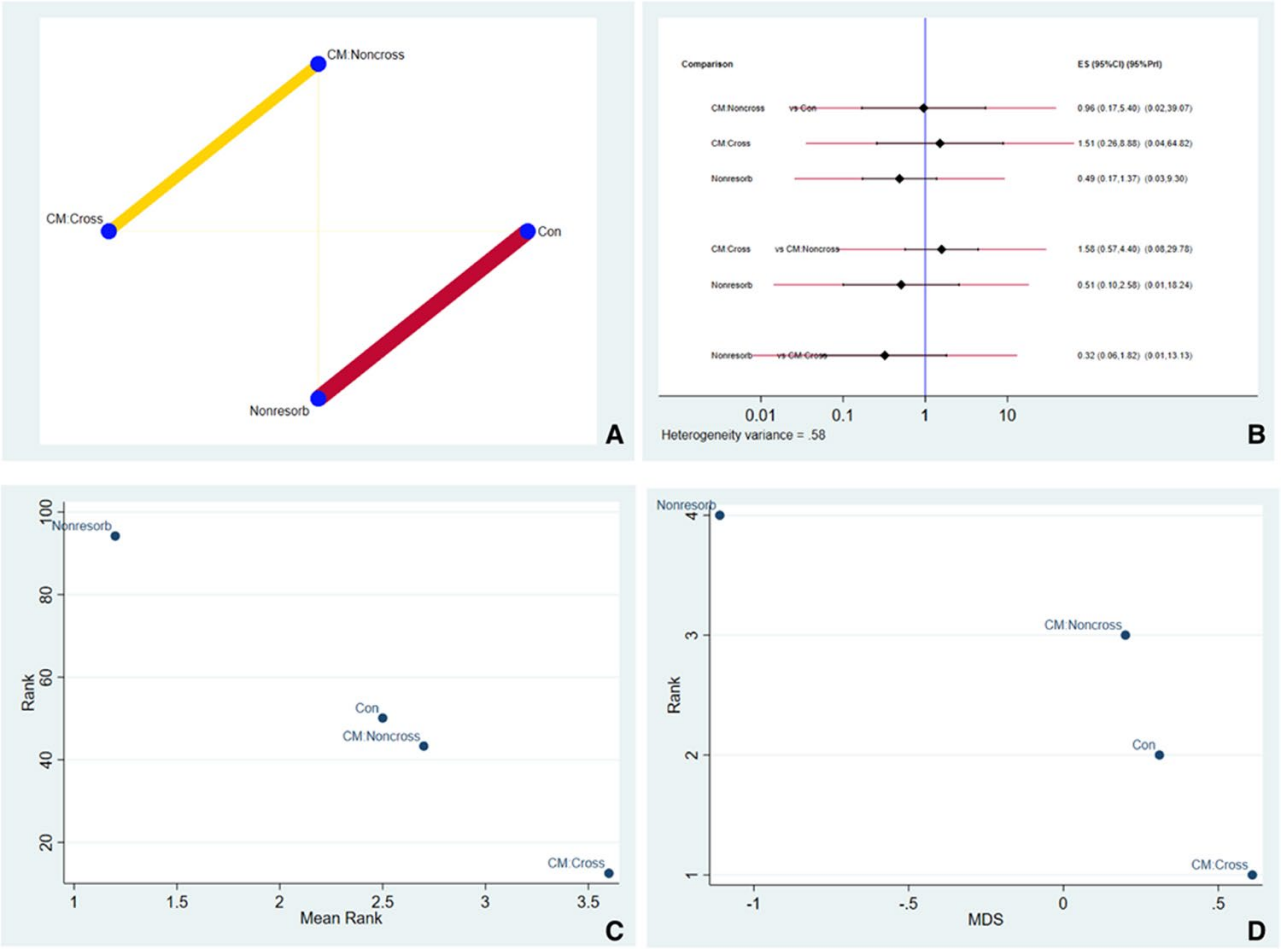
**A** Illustrates the network geometry plot for vertical dimensional changes. **B** Predictive interval and confidence interval plot for vertical dimensional changes. **C** The surface under the cumulative ranking curves (SUCRA) that expresses the percentage of effectiveness/safety each treatment has compared to an “ideal” treatment ranked always first without uncertainty. **D** Multidimensional scale ranking demonstrating the ranking of the sealing material in vertical dimensional outcomes based on dissimilarity

**Fig. 4:**
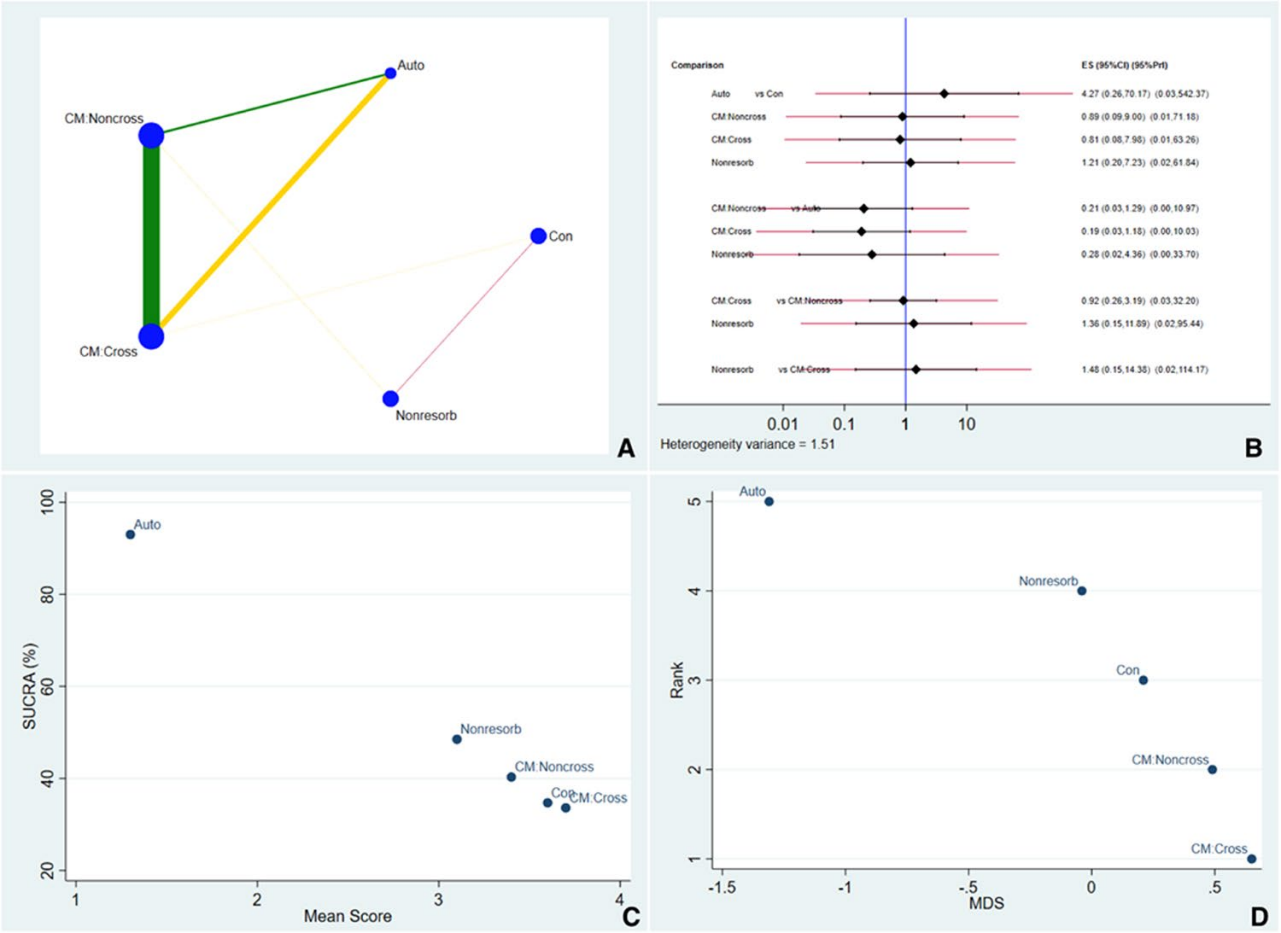
**A** Illustrates the network geometry plot for horizontal dimensional changes. **B** Predictive interval and confidence interval plot for horizontal dimensional changes. **C** The surface under the cumulative ranking curves (SUCRA) that expresses the percentage of effectiveness/safety each treatment has compared to an “ideal” treatment ranked always first without uncertainty. **D** Multidimensional scale ranking demonstrating the ranking of the sealing material in horizontal dimensional outcomes based on dissimilarity

Fig. 3A shows the network geometry plot for the overall vertical dimensional changes. The size of the blue circles is proportional to the sample size for each sealing biomaterial. The thickness of the lines connecting two circles is proportional to the number of studies comparing two treatments. Resorbable non-crosslinked collagen membranes (CM:NonCross) were the most highly represented coverage biomaterial utilized. Fig. 3B shows the effect size and confidence intervals for each comparison. Values to the left of the vertical blue line indicate favorable outcomes for the test group (e.g., in the top group, CM:NonCross, CM:Cross, and Nonresorb were test groups, and Con was the control group). Confidence intervals (black horizontal lines) not crossing the blue line indicate significant differences relative to the control. For example, in Fig. 3B, the first comparison indicates an insignificant advantage of CM:NonCross over the control group, while in the second comparison, the control group has an insignificant advantage over CM:Cross (CM:Cross more likely to perform better in future clinical studies). Fig. 3C illustrates the SUCRA ranking and suggests CM:Cross ranked higher in performance in vertical outcomes. Fig. 3D illustrates multidimensional ranking of the different sealing types based on dissimilarity of the material. The IF for vertical dimension outcome was illustrated in Fig. S4A and Fig. S4B. The ROB between comparisons was illustrated in Fig. 4C.

#### Horizontal outcomes

Fig. 4A shows the network geometry plot for overall horizontal dimensional changes. The most frequent comparison was between CM:NonCross and CM:Cross. Fig. 4B shows that all comparisons displayed an insignificant difference between sealing materials, as all confidence intervals overlapped with the vertical blue line. Autologous soft tissues and non-resorbable membranes are more likely to perform better in future clinical studies. Based on Fig. 4C, it illustrates the SUCRA ranking and suggestive of autologous soft tissue material ranked higher in performance in horizontal outcome. Fig. 4D illustrates multidimensional ranking which ranked the sealing material based on the dissimilarity of the material. The IF for horizontal outcome was illustrated in Fig. S5A and Fig. S5B and ROB between comparisons was illustrated in Fig. S5C.

#### Histomorphometric outcomes

**Fig. 5:**
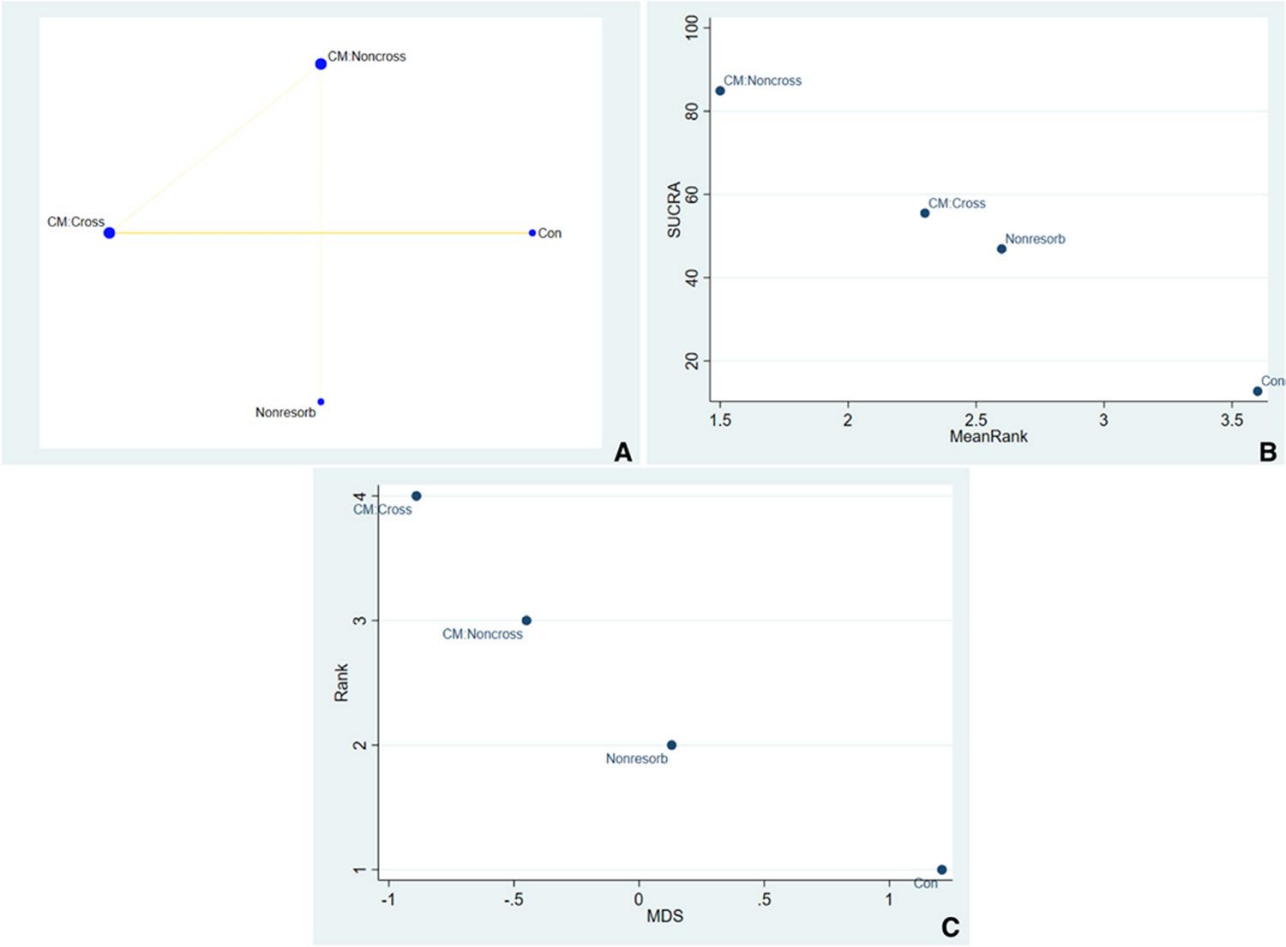
**A** Illustrates the network geometry plot for histomorphometric changes. **B** The surface under the cumulative ranking curves (SUCRA) that expresses the percentage of effectiveness/safety each treatment has compared to an “ideal” treatment ranked always first without uncertainty. **C** Multidimensional scale ranking demonstrating the ranking of the sealing material in histomorphometric outcomes based on dissimilarity

Histomorphometric NMA analysis included studies of Arbab et el., Lim et al., and Engler-Hamm et al. [11, 19, 23]. Network geometry plot for histomorphometric analyses is illustrated in Fig. 5A. There are only three studies and hence were not able to predict which sealing material would perform better in future clinical studies. However, we used forced function in the STATA commands to generate ranks for the sealing materials. The SUCRA ranking suggests that CM:Noncross ranked highest in performance (Fig. 5B). Fig. 5C illustrates the multidimensional scale ranking based on dissimilarity between two competing treatments. This gives a picture on the level of dissimilarity in individual dataset. The ROB between comparisons was illustrated in Fig. S6.

## Discussion

The use of bone graft and coverage biomaterials during ARP has been shown to result in superior treatment outcomes compared with unassisted socket healing regarding preservation of ridge width and height.

The great heterogeneity of the included studies prevented a balanced comparison among these articles and made it difficult to consider the contribution of all the confounding factors towards treatment outcomes.

In this review, the authors included articles in which comparisons are done between spontaneous healing versus spontaneous healing associated to the use of a membrane [Mandarino e Lekovic]: the reader may speculate that the shrinkage of horizontal bone could be because of the lack of bone graft material and not because of the sealing material. The authors are convinced that this comparison is interesting because in comparing no material with or without a membrane, the difference between the two groups will be due to the membrane/soft tissue and not to the bone graft itself.

The standard pairwise meta-analysis comparing the same two coverage biomaterials could only aggregate the data from two studies per outcome. For the comparison between resorbable membranes and the control group, all the forest plots found a statistically significant difference in favor of the groups using resorbable membranes, with no statistically significant heterogeneity. In the studies evaluating dimensional changes, the test and control groups were filled with the same graft material (collagenated deproteinized bovine bone matrix in the study by Lim et al. [19] and no grafting material in the study by Lekovic et al. [24]), suggesting that any observed differences were likely due to the choice of coverage biomaterial.

For the comparison between crosslinked and non-crosslinked collagen membranes, both plots found a statistically significant difference in favor of the latter membrane type. It is well-known that the crosslinked membranes, even if with a better resistance to degradation, are less biocompatible than non-crosslinked ones [26, 27]. Moreover, the crosslinked membranes performed statistically significantly better if non-exposed [26]. For this reason, it might be speculated that, mostly in thin gingival phenotypes, the lower biocompatibility and the possible consequent exposure of the membrane could play a role in the lower clinical performance of the crosslinked membranes.

In order to compare results obtained with different coverage biomaterials, a NMA employing direct and indirect comparisons was performed. The network geometry plot for the overall vertical changes showed a moderate level of reliability (yellow line) for the comparison between non-crosslinked resorbable membranes and the control group. In contrast, a lower level of reliability was found for the comparison between non-resorbable membranes and the control.

The same plot was used to compare the overall horizontal changes. In this case, a high level of reliability (green line) was found for the comparison between crosslinked and non-crosslinked resorbable membranes and, to a lesser extent, between non-crosslinked resorbable membranes and autogenous soft tissue grafts. A moderate level of reliability (yellow line) was found for the comparison between non-crosslinked resorbable membranes and the control group. The width of the green and yellow lines in this latter plot was related to a high degree of homogeneity among included studies and a minor risk of bias. Reliability depends not only on the amount of evidence (number of studies and number of patients) but also on the heterogeneity within study results and the variability between different studies (e.g., size of the standard deviations).

In terms of the predictive interval plot for the socket preservation network, differences between horizontal and vertical changes were found. None of the comparisons showed a statistically significant difference. For horizontal changes, the autogenous group was associated with superior outcomes compared to crosslinked and non-crosslinked resorbable membranes, although this comparison was not statistically significant. Regarding vertical changes, crosslinked membranes showed poorer results both in terms of the number of studies and the clinical outcome. The sealing biomaterial which had the highest probability of preserving the horizontal dimension was the autogenous group, followed by non-resorbable membranes. These results must be taken cautiously as they were derived from a limited number of articles with a low sample size. Obviously, more studies with larger numbers of patients are necessary to confirm these findings.

From a histological point of view, only 5 studies were included [11, 12, 19, 22, 23]; the others did not present a histologic analysis. However, it must be highlighted that a substantial inconsistency was found in the 5 selected studies. In fact, only Hassan et al. [12] made a comparison between test (amnion-chorion membrane (ACM)) and control groups (d-PTFE) in terms of percentage of newly formed bone.

Specifically, the use of an amnion-chorion membrane seemed to provide a better quality of newly formed bone, with a greater amount of new osteoid formation (8.31% vs 3.5%) and a lower number of graft particles (6.76% vs 12.31%) compared to the control group treated with an d-PTFE membrane. This might suggest that amnion-chorion membrane produced a relatively more rapid bone turnover rate. Other authors presented only mean values without a real comparison between different groups. For this reason, the network plot demonstrated inconsistency among studies which prevented further statistical analysis.

The main limitations of the present review are the small number of studies included and a relatively high heterogeneity among studies, in terms of treatment approaches, materials used, follow-up duration, and outcome assessment. Therefore, the findings of the present NMA must be interpreted cautiously.

## Conclusion

According to the results of the network meta-analysis, only some trends regarding the use of different sealing materials can be found: no specific sealing techniques and/or biomaterials can be recommended over another in the context of ARP due to a lack of sufficient data. Only some trends can be highlighted, as shown in the results. The limited number of studies comparing ARP with and without biomaterials to seal the socket suggests that the application of a membrane is associated with superior results in terms of preservation of alveolar ridge dimensions. The RCTs studying this topic with larger sample sizes are needed in order to better elucidate the effects of different coverage biomaterials on ARP treatment outcomes.

## Supplementary Information

Figure S1A (30.3 KB)

Figure S1B (29.5 KB)

Figure S2 (29.7 KB)

Figure S3A (30.6 KB)

Figure S3B (28.7 KB)

Figure S4A (153 KB)

Figure S4B (158 KB)

Figure S4C (190 KB)

Figure S5A (177 KB)

Figure S5B (204 KB)

Figure S5C (225 KB)

Figure S6 (178 KB)
