# Supplementary figures and images for: Sealing materials for post-extraction site: a systematic review and network meta-analysis
Source: Clin Oral Investig. 2021 Nov 25;26(2):1137–54. doi: 10.1007/s00784-021-04262-3 (PMC8816783; doi:10.1007/s00784-021-04262-3)

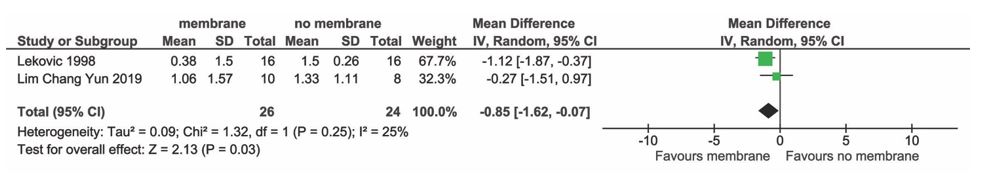

Supplement: Supplementary file 1 — (30.3 KB) [file 784_2021_4262_MOESM6_ESM.jpg]

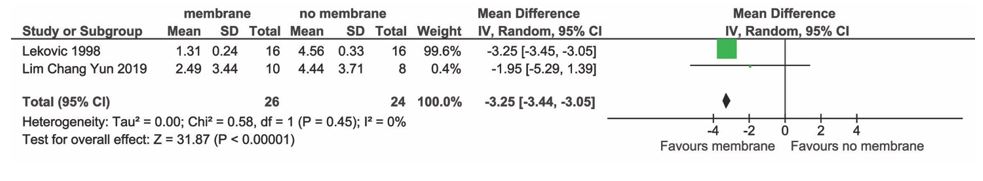

Supplement: Supplementary file 2 — (29.5 KB) [file 784_2021_4262_MOESM7_ESM.jpg]

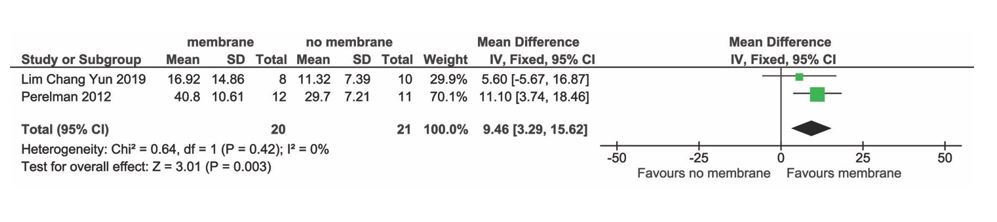

Supplement: Supplementary file 3 — (29.7 KB) [file 784_2021_4262_MOESM8_ESM.jpg]

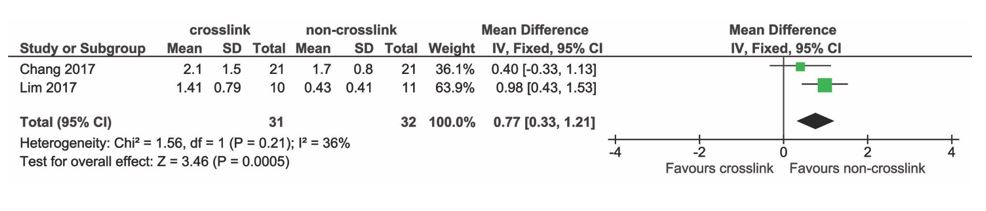

Supplement: Supplementary file 4 — (30.6 KB) [file 784_2021_4262_MOESM9_ESM.jpg]

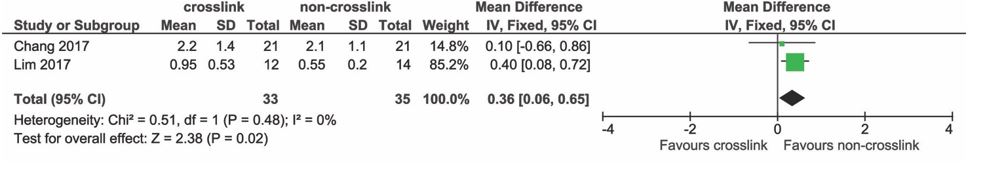

Supplement: Supplementary file 5 — (28.7 KB) [file 784_2021_4262_MOESM10_ESM.jpg]

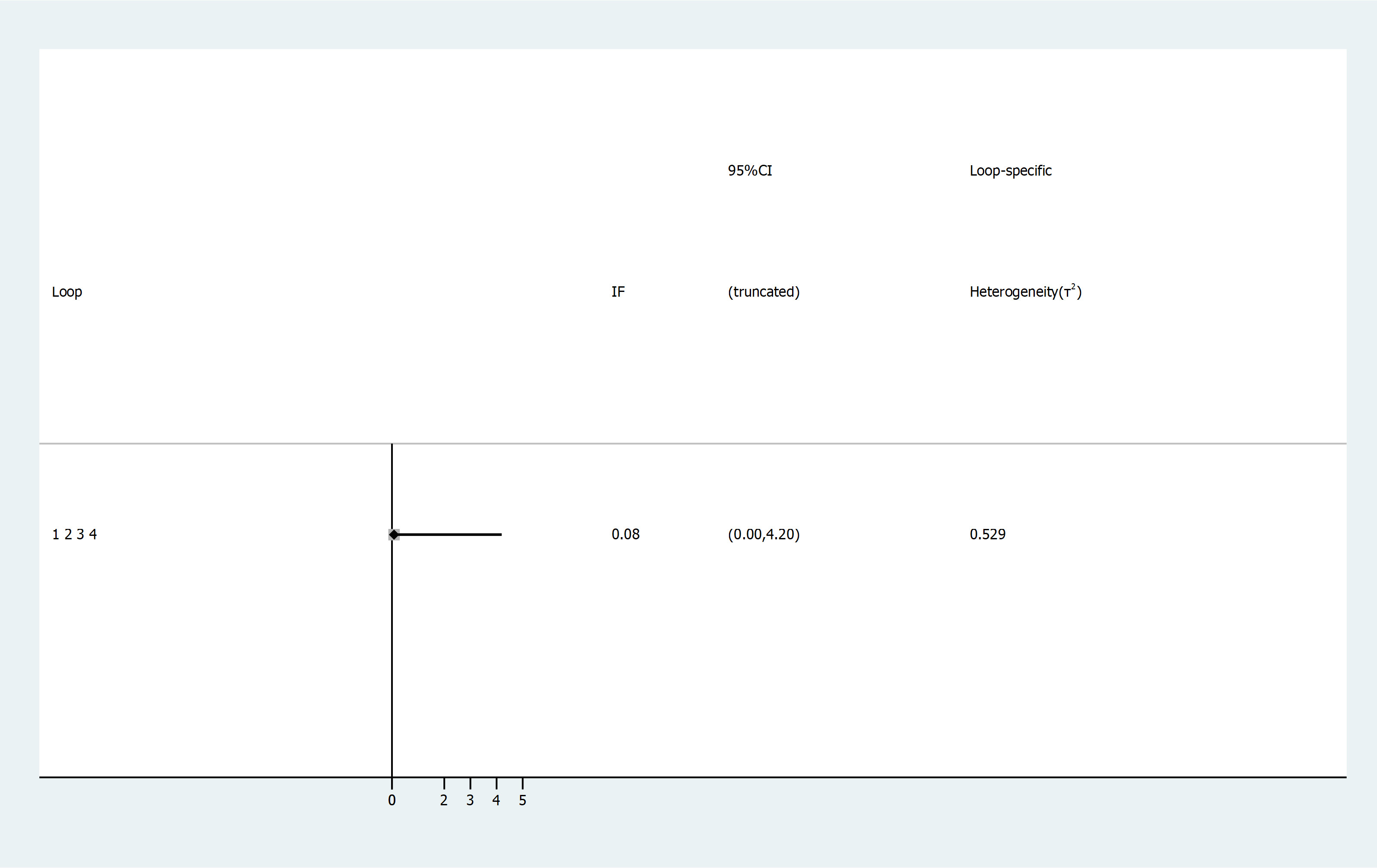

Supplement: Supplementary file 6 — (153 KB) [file 784_2021_4262_MOESM11_ESM.jpg]

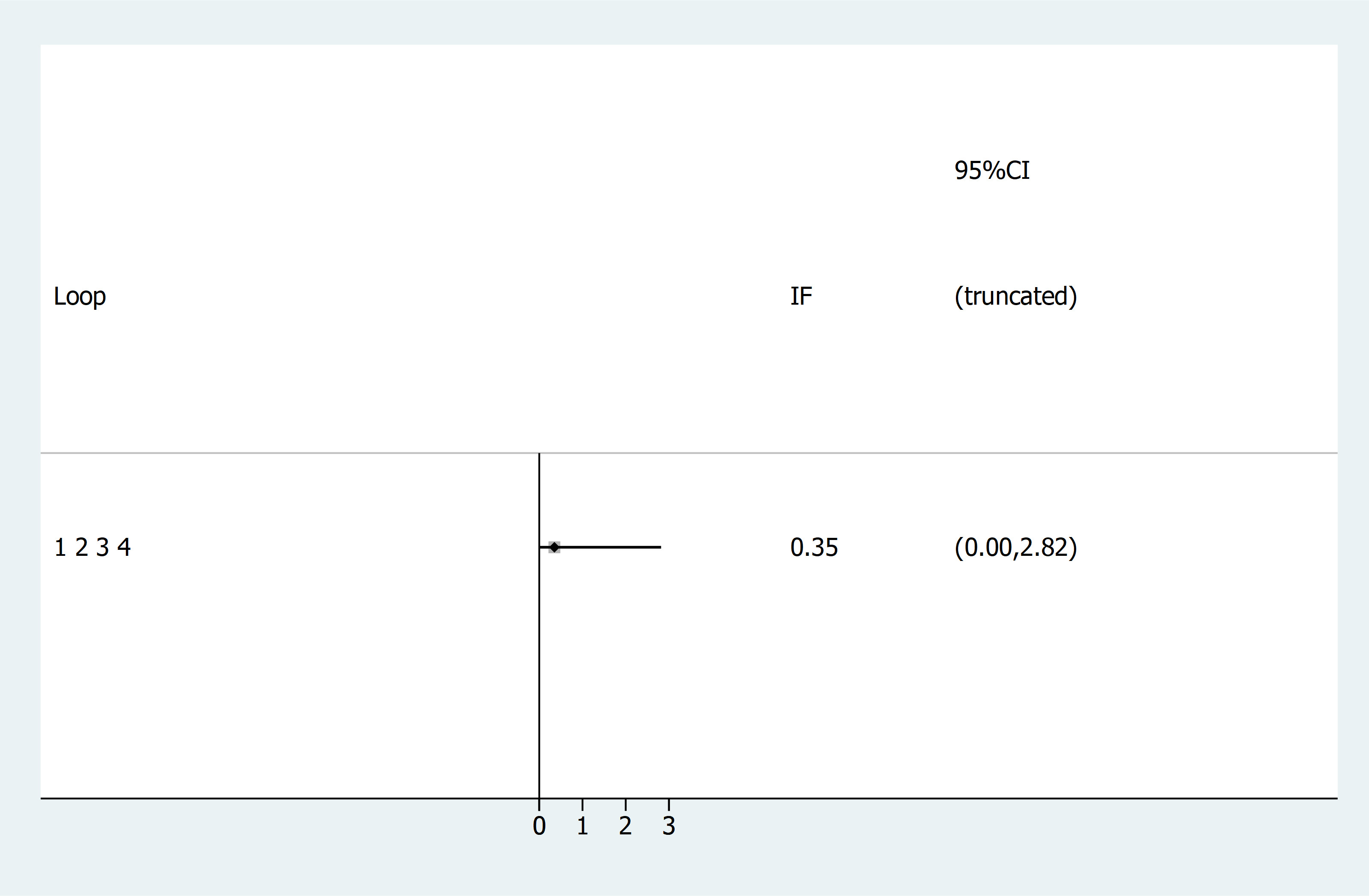

Supplement: Supplementary file 7 — (158 KB) [file 784_2021_4262_MOESM12_ESM.jpg]

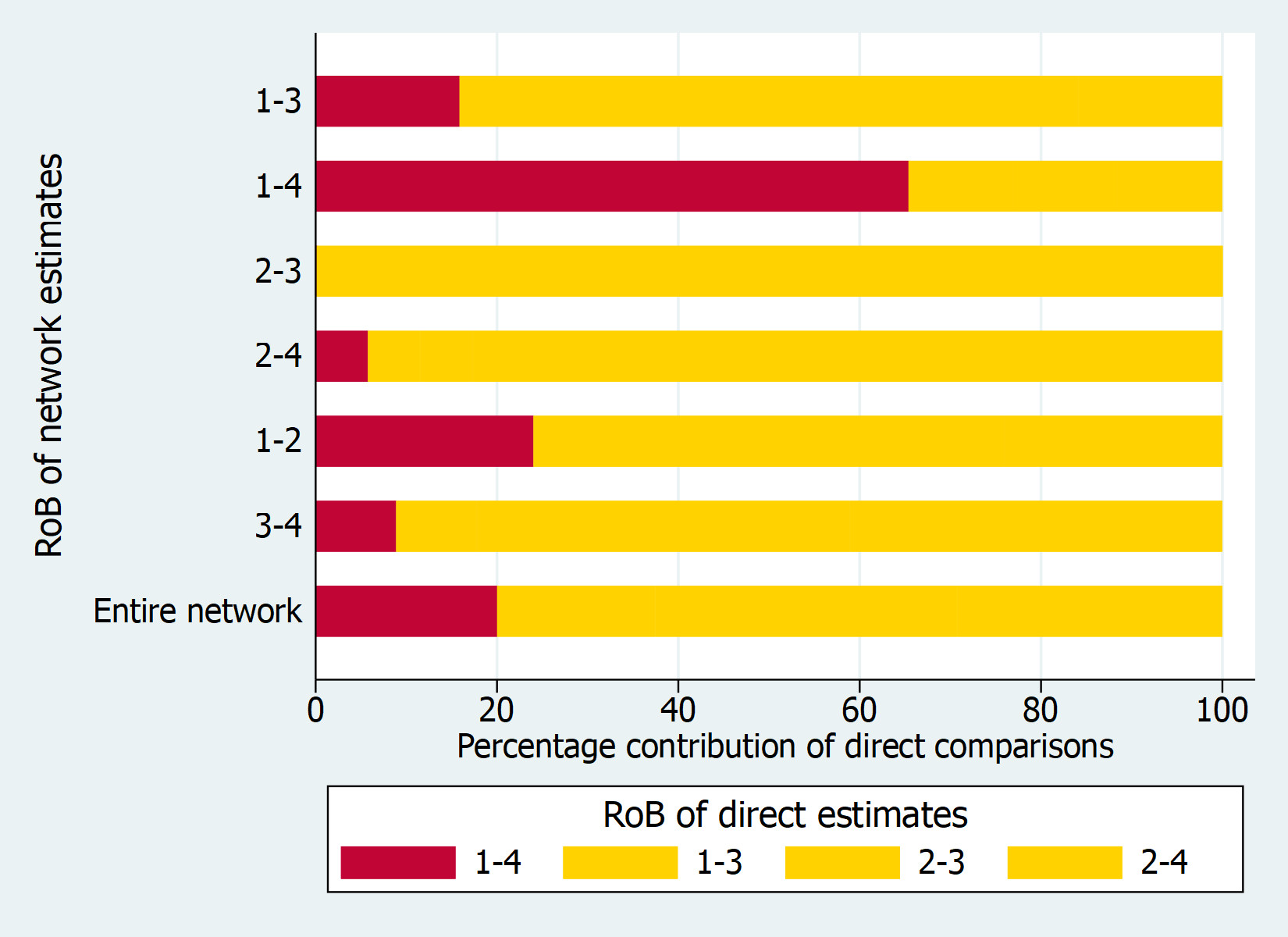

Supplement: Supplementary file 8 — (190 KB) [file 784_2021_4262_MOESM13_ESM.jpg]

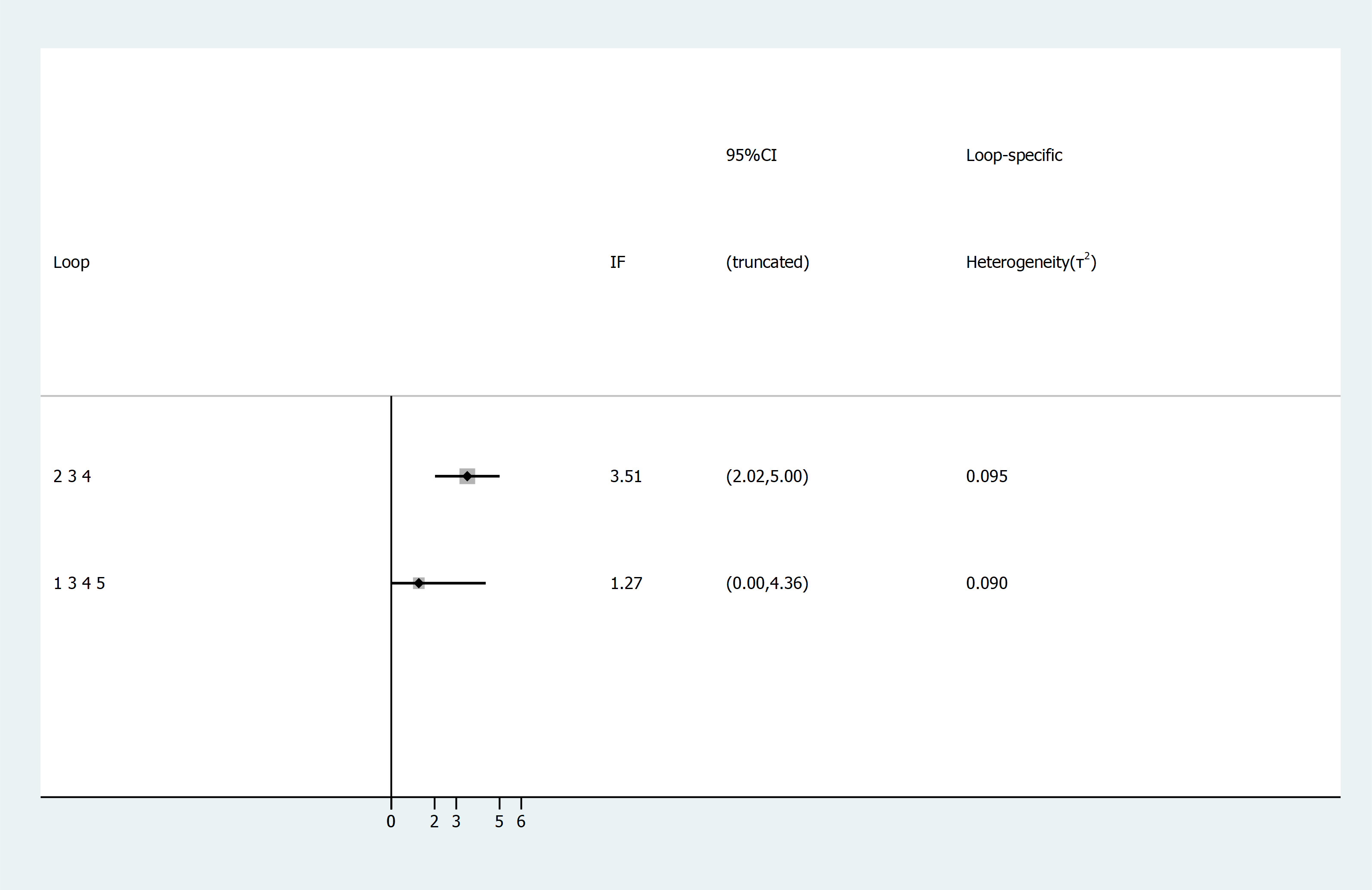

Supplement: Supplementary file 9 — (177 KB) [file 784_2021_4262_MOESM14_ESM.jpg]

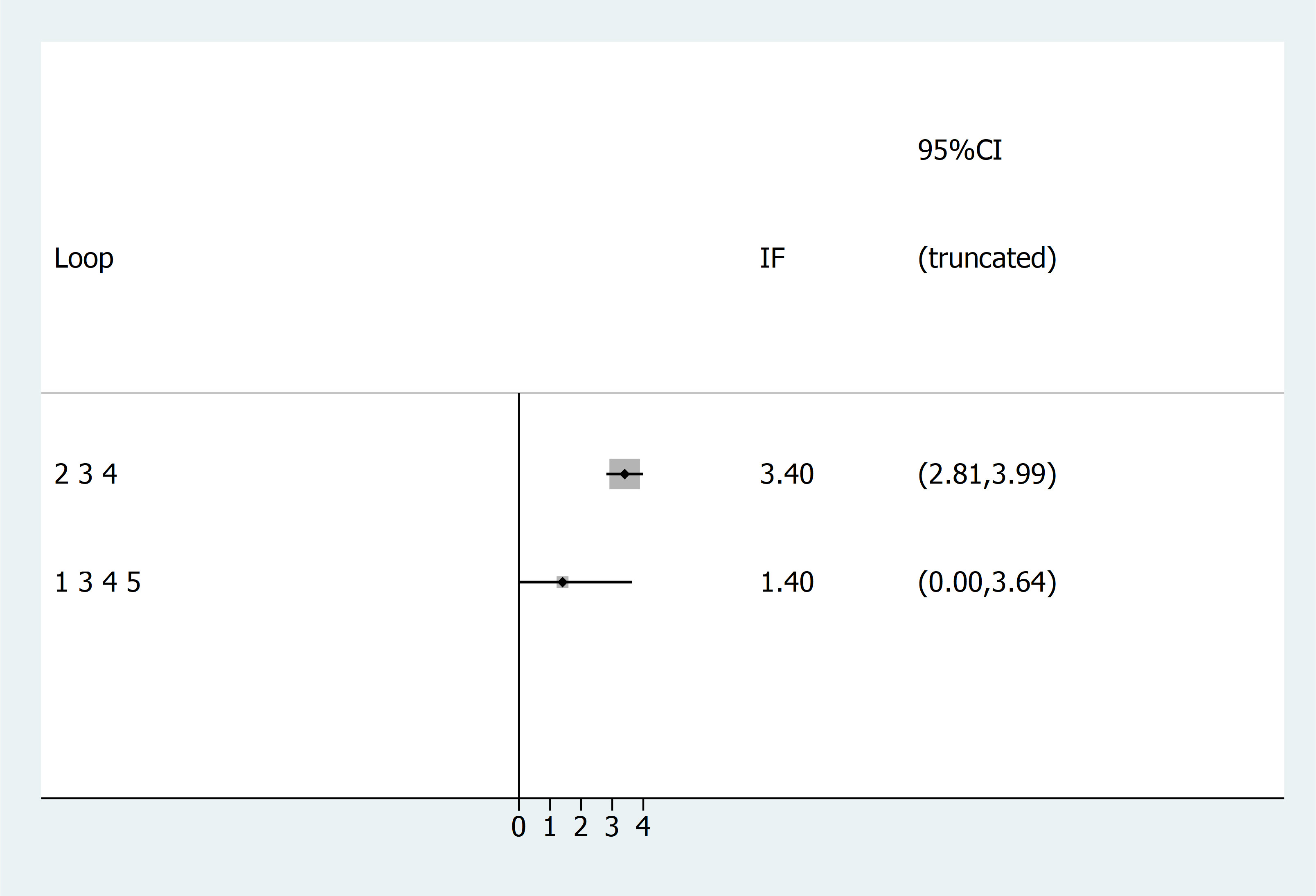

Supplement: Supplementary file 10 — (204 KB) [file 784_2021_4262_MOESM15_ESM.jpg]

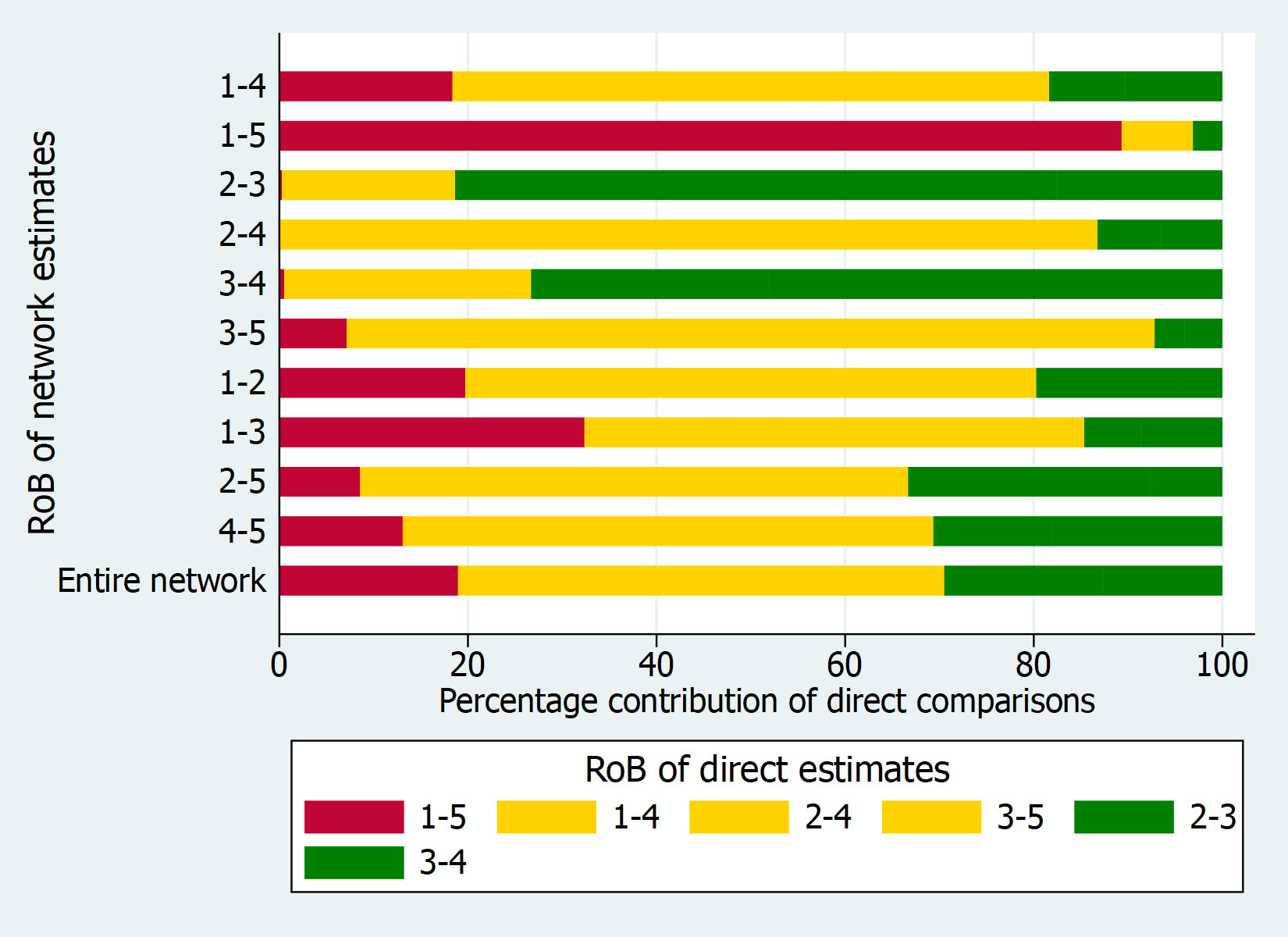

Supplement: Supplementary file 11 — (225 KB) [file 784_2021_4262_MOESM16_ESM.jpg]

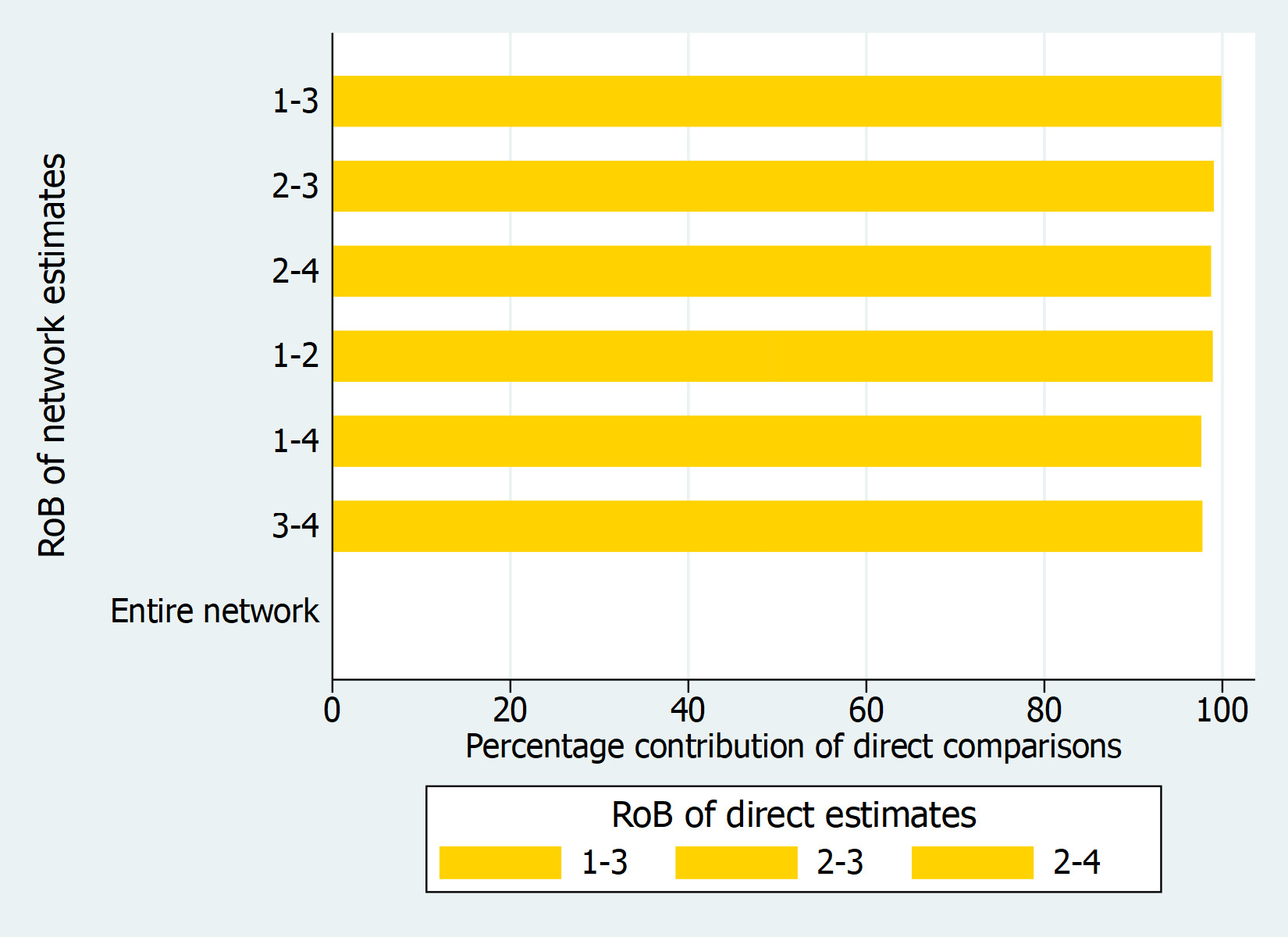

Supplement: Supplementary file 12 — (178 KB) [file 784_2021_4262_MOESM17_ESM.jpg]
